# A Critical Appraisal of the Effect of Gonadotropin-Releasing Hormon Analog Treatment on Adult Height of Girls with Central Precocious Puberty

**DOI:** 10.4274/jcrpe.2017.S004

**Published:** 2017-12-30

**Authors:** Abdullah Bereket

**Affiliations:** 1 Marmara University Faculty of Medicine, Department of Pediatrics, Division of Pediatric Endocrinology, İstanbul, Turkey

**Keywords:** Central precocious puberty, gonadotropin-releasing hormon analogues, treatment, final height, adult height, growth, triptoreli, leuprolide

## Abstract

Central precocious puberty (CPP) is a diagnosis that pediatric endocrinologists worldwide increasingly make in girls of age 6-8 years and is mostly idiopathic. Part of the reason for increasing referral and diagnosis is the perception among the doctors as well as the patients that treatment of CPP with long-acting gonadotropin-releasing hormon analogues (GnRHa) promote height of the child. Although, the timing and the tempo of puberty does influence statural growth and achieved adult height, the extent of this effect is variable depending on several factors and is modest in most cases. Studies investigating GnRHa treatment in girls with idiopathic CPP demonstrate that treatment is able to restore adult height compromised by precocious puberty. However, reports on untreated girls with precocious puberty demonstrate that some of these girls achieve their target height without treatment as well, thus, blurring the net effect of GnRHa treatment on height in girls with CPP. Clinical studies on treatment of girls with idiopathic CPP on adult stature suffers from the solid evidence-base due mainly to the lack of well-designed randomized controlled studies and our insufficiencies of predicting adult height of a child with narrow precision. This is particularly true for girls in whom age of pubertal onset is close to physiological age of puberty, which are the majority of cases treated with GnRHa nowadays. Heterogeneous nature of pubertal tempo (progressive vs. nonprogressive) leading to different height outcomes also complicates the interpretation of the results in both treated and untreated cases. This review will attemp to summarize and critically appraise available data in the field.

## INTRODUCTION

Gonadotropin-releasing hormone analogues (GnRHa) are the treatment of choice for nearly four decades in children with central precocious puberty (CPP) (1). Treatment effectively suppresses hypothalamo-pituitary-gonadal axis, which results in arresting early and accelerated activation of sex hormone synthesis, progression of secondary sexual characteristics and undue maturation of the skeletal development, thus meeting the aims of the treatment, which are 1) to prevent potential psycological problems related to early pubertal development, and 2) to restore genetic growth potential otherwise compromised by sex-hormone-driven premature closure of bone growth plates.

Although the majority of the studies in the field suggests beneficial effects of treatment, there have been ongoing uncertainties about the achievement of both aims of the treatment due to methodological limitations of the present studies. This review wil focus on the effect of GnRHa treatment on height outcome in girls with CPP.

Uncertainties about the benefits of GnRH analog treatment on growth of children with CPP comes from the fact that there is no randomised controlled study on this respect. Some of the studies in this field compare treatment group with a historical control groups which are reported decades ago, include limited number of subjects and heterogeneous with respect to nuances of pubertal development. Some studies have their own untreated control group (but not randomised) which brings biases to the interpretation of the data. Finally, many studies are comparing the achieved adult height with predicted adult height (PAH) at initiation of treatment which suffers from the limitations of our ability to assess bone age (BA) and predict adult height precisely, and disregard the genetic height potential of the child.

### The Relationship Between Height and Timing of Puberty

It has been known for a long time that physiological variations in the time of pubertal development has an effect on statural height. Shangold et al (2) evaluated the relationship between recalled menarcheal age and adult height, in 425 women. After exclusion of those in whom menarche occurred after age 16, the overall linear regression equation for the remaining 416 patients, height = 153.95 + 0 .7353 x (age of menarche), was significant. Average height in women who had menarche at age 9 was 159.5±6.5 whereas those with menarche at age 11-13 yrs was 163 cm. Overall the data suggested that menarcheal age significantly correlates with adult height as an independent variable (2).

A large longitudinal study on American girls also evaluated the effect of timing of spontaneous puberty on height was indicated a higher adult height in girls with late (>12.9 years) versus early (<11.7 years) age at menarche. The median difference was of 2.6 and 1.7 cm in white and black girls respectively (3). A recent Korean study of 4218 post-menarcheal girls between the ages of 16 and 18 years reported mean heights of early (9.9±0.2 years), average (12.5±0.9 years) and late (15.1±0.3 years) menarche groups as 160.4±5.2 cm, 161.8±4.9 cm, 162.3±4.7 cm respectively p=0.001) (4).

In contrast to above studies, a recent longitudinal study from Thailand followed 104 girls with breast development at 7.0-9.0 years. Despite the average age at menarche was early (10.2±0.9), their near final height obtained at 12.6±0.4 years was 154.0±4.9 cm, which was similar to their average target height (TH) of 153.1±4.8 cm (5).

It can be concluded from above mentioned studies that “early” puberty within the currently accepted physiological range has “if any” a very small (2-4 cm) effect on adult height reached, an observation consistent with none to very small height gain achieved in GnRH analog treatment of girls with “early” puberty (6,7,8). However, “truely” precocious puberty starting at a very young age is expected to result in more loss in height potential depending on the age at start and the tempo of puberty. Precise estimation of the height loss caused by precocious puberty is difficult to estimate because of the scarcity and imperfections of data in that respect.

### Height Outcome in Girls with Precocious Puberty without Treatment

Historical series of untreated patients (Table 1) reported mean heights of 152 cm in girls and 156 cm in boys, a loss of ~10 cm in girls and 20 cm in boys (9,10,11,12,13,14). However, these data should be interpreted very cautiously. First of all, those data come from a limited number of patients from the 1950s and 1960s with cases that are very severe and early onset CPP, with cases due to organic reasons constituting the great part of it. Thus, more severe than the average patient treated today. In fact, in most of these historical series, there was a negative correlation between the age of onset of precocious puberty and adult height, confirming the poor height prognosis of the most severe and early cases. Furthermore, some of the untreated patients with organic CPP may have had growth limitation due to factors associated with their central nervous system disorder, such as growth hormone (GH) deficiency. Secondly, these were not large series, especially for the figure of boys which derived from total of 38 untreated boys in total of four studies (9,10,11,12). Lastly, these studies do not take into account the secular increase in height.

In one of the early studies, Paul et al (11) compared their treated patients with untreated subjects from the literature (9,10,13,14). The final height of treated females was 160.5±6.6 cm whereas matched untreated historical females had a height of 152.7±8.6 cm (difference of treated vs. untreated 7.8 cm). Although treated girls’ mean final height was still -1 standard deviation (SD) below mean midparental TH, this was better compared to untreated ones who had height -2.4 SD below TH. Further classification of the patients according to age revealed that untreated girls who were <5 years of age had a mean final/near final height of 150.2±7.6 cm whereas those treated reached 164.3±7.7 (difference of treated vs. untreated 14.1 cm). Untreated girls who were >5 years of age had a mean final height of 153.4±8.4 cm whereas those treated reached 157.6±6.6 (difference of treated vs. untreated 4.2 cm).

Kletter and Kelch (15) reviewed this matter in 1994. They found more modest height gains in treated girls compared to untreated girls (6.5 cm and 0.5 cm in <6 yrs and >6 yrs respectively). However, when they compared patients with their TH, the effect of GnRHa treatment on height was much less (only 2.7 cm in whom puberty started before 6 years of age and no height gain in those >6 yrs). The authors concluded that treatment with GnRH agonist analog does not significantly alter the final adult height of girls with idiopathic CPP whose age at diagnosis is greater than 6 years.

The obvious difference between the conclusion of these studies might arise from the heterogeneity of the subjects in regard to TH, and the tempo of puberty in the subjects (both treated and untreated). As most untreated patients in these series were seen before the introduction of computed tomography it is quite possible that some who had a small intracranial lesion, for example a small hypothalamic hamartoma, were included in this untreated “seemingly” idiopathic CPP groups.

Nevertheless, those studies with untreated control groups (Table 2) (11,15), as well as later studies without control groups (Table 3) (16,17), confirmed that age is an important determinant of treatment outcome and that earlier the age of onset of CPP, the worst is the height outcome if left untreated. Thus, earlier the onset of treatment, height gain achieved by the GnRHa treatment is bigger.

However, unlike historical untreated cohorts mentioned above, some studies afterwards reported final height of untreated girls with CPP demonstrated less, or no decrease in height compared to their TH. Bar et al (18) reported final height data of 20 and near final of 7, girls with idipathic CPP. The appearance of breast tissue occurred at 5.6±1.6 years; the first evaluation was performed at 7.0±2.4 years. Six children were less than 6 years of age at the time of the initial evaluation. Although the mean BA was 8.4±3 years, one third of the girls had a BA at least 2 years (range, 2 to 3.7 years) greater than their chronologic age. The mean age of menarche was 10.5 years which was 4.9±2.4 years (range, 3 to 13 years) after thelarche. Despite that, adult height was normal in 90% of girls (mean, 161.4±7.7 cm). Although parental heights were not available in this study, mean final height of the untreated girls with ICCP were only slightly less than healthy average American women 163.8 cm.

In another study, untreated control group consisted of 10 girls with idiopathic precocious puberty who, at their parents’ request, were not treated. Mean age at the onset of pubertal signs was 6.05±0.3 years. There was no significant difference between final height of treated (152.4±1.4 cm) and untreated (149.5±2.0 cm) girls. Final height was significantly lower than TH in both treated (with ciproteron) (155.1±0.9 cm; and untreated (156.4±1.3 patients, but the mean height of treated patients is nearer to TH than that of untreated ones (19).

In a similar study, Kauli et al (20) reported final height of 28 untreated girls with ICCP. Fourteen of them had a slow course of puberty and reached final height of 160.2±7.1 (their TH was 159.5±6.6 cm); the other half (14/28) had an accelerated course of puberty with a final height well below TH (final height 150.8±4.3, TH 159.2±5.9 cm) and in most cases (14/28) below the height SD score (SDS) of both parents.

Obvious differences in the height outcome of untreated patients in different studies (historical cohorts versus more recent cohorts) reflect the heterogeneity of the patients in regard to pubertal hormonal activation. As in Kauli et al’s (20) study, it has been shown in several series that in a subgoup of the girls presenting with what appears to be idiopathic CPP, will either have stabilization or very slow progression in their pubertal signs. Progression of hormonal activation is somewhat slower in these girls and the final heights are not compromised. The BA is typically not as advanced compared with children with true CPP, and serum lutenizing hormone (LH) concentrations are within the pre- or early-pubertal range, indicating that the hypothalamic-pituitary-gonadal axis is not fully activated. GnRH stimulation test in these children demonstrate a follicle-stimulating hormone (FSH) dominant response. These children are considered to have slowly progressive form of CPP.

Palmert et al (21) reported 12-yr follow-up of 20 patients who initially presented with unsustained or slowly progressive puberty by the presence of one or more of the following findings: menses, pubic hair, accelerated growth velocity, and/or BA greater than 2 SD above chronological age. None of the 20 patients had a pubertal response to exogenous GnRH; (by that time with an radioimmunoassay LH increase of less than 25 IU/L above baseline and a peak FSH greater than or equal to the peak LH in response to exogenous GnRH). Thus, at that time, these girls were not considered candidates for long term pituitary-gonadal suppression with a GnRH agonist. Seventy percent of those patients experienced cessation of their early pubertal development, whereas the remainder reported a slowly progressive course. Those with a slowly progressive course were significantly older than those with an unsustained course [mean age of thelarche, 6.1 vs. 3.4 yr; age of pubarche, 6.0 vs. 4.0 yr. They also had more advanced skeletal maturation (BA, 10.2 vs. 7.3 yr; at the time of evaluation. Both groups, however, had similar outcomes with respect to linear growth and young adult reproductive function. On the average, the study patients reached their genetic targets for final height (mean final height, 165.5±2.2 cm; mean genetic TH, 164.0±1.1 cm; p 5 NS). The average age of menarche was 11.0±0.4 yr.

Léger et al (22) also followed 9 patients (mean age 6.5 years, range 4.8-7.7 years) with a slowly progressing variant of CPP without treatment; final height (161.8±4.6 cm) was similar to the pre-treatment predicted height (163.1±-6.2 cm) and was not significantly different from TH (161.0±5.9 cm).

Table 4 summarizes height outcome of girls with untreated CPP (slowly progressive, milder, or older onset) patients in different series. Final height-TH ranged between -6.8 cm to 1.6 cm. On average, final height was -4.4 cm shorter than TH in six studies (15,18,19,20,23,24) but similar to TH in the remaining seven studies (20,21,22,25,26,27,28). Thus, it can be concluded that the different height outcome of girls with untreated idiopathic CPP in various studies are due to the fact that natural course of precocious puberty differs from one subject to another, i.e. some are more progressive hence have unfavorable outcome whereas some are slowly progressive hence favorable outcome in regards to final height.

### Identification of Girls with Progressive Central Precocious Puberty

There is not enough data about the ratio of progressive vs. nonprogressive precocious puberty among girls who develop breast development before 8 years of age. Kaplowitz (29) reported 9% of true precocious puberty in 104 children referred for any signs of early puberty, whereas this ratio was higher (47%) in another US study of 223 girls referred for precocious puberty between ages 7 and 8 (white girls) or 6 and 8 (black girls) (30). Mogensen et al (31) reported nearly 20% true precocious puberty, among 449 girls referred for early pubertal signs. All of these cohorts included all variants of early pubertal development including premature thelarche, premature adrenarche and early normal variants (those >age 8 yrs). However, we have recently reviewed 236 girls who presented with breast development between ages 4-8 years (thus excluding premature adrenarche, thelarche variant etc.). 59% of these girls were eventually diagnosed with true precocious puberty and given GnRHa treatment (20). This was nearly 34% in Mogensen et al’s (32) series after exclusion of other variants.

Although the mechanism of why puberty is nonprogressive in certain girls is unknown, some clinical features have been proposed to help identifying those who will likely to progress rapidly, although specifity and sensitivity of these criteria varies greatly (33,34,35,36,37,38,39,40) (Table 5). Along with clinical and anthropometric criteria, GnRH-stimulated LH levels of 5 IU/L have been suggested to mark the beginning of puberty using one modern immunochemiluminometric assays (34,35). Stimulated LH limit of 5 IU/L to define CPP was found to have specificity of (77%), and sensitivity of (95%) (36). In one study, randomly measured LH values of 0.3 IU per liter and above were reported to be 100% specific for peak values above 5 IU per liter (37). However, in young children (2-4 years) gonadotropin levels are normally high and therefore LH (basal or peak) should be carefully interpreted in this age group (38). In the consensus report on the use of GnRHa treatment, mentioned values for uterine length range from 3.4 to 4.0 cm (1). The cutoffs for a pubertal ovarian volume range between 1 and 3 mL (volume: length x width x height x 0.5233) (39). A uterine volume greater than 2.0 mL has been reported to have 89% sensitivity and specificity for precocious puberty (40).

As distinguishing progressive form of CPP from nonprogressive forms is important for therapeutic decision-making, the Consensus Conference Group has recommended that progressive pubertal development be documented for 3-6 months before starting GnRHa treatment This observational period may not be necessary if the child is at or past Tanner stage 3 (breast), particularly with advanced skeletal maturation (1).

In addition to above mentioned anthropometric and clinical criteria, we should be aware of certain risk groups in whom precocious puberty is likely to be progressive. These are, family history for precocious puberty, being born small for gestational age (SGA), and adopted children. One has to carefully follow these children when they develop breast development early, as they likely to have progressive precocious puberty. Familial forms of precocious puberty tend to be more progressive than those of sporadic ones. Comparison of 43 familial cases among the total cohort of 156 (147 girls and 9 boys) cases of idiopathic CPP, it was found that the familial group had lower maternal age at menarche than the sporadic group (mean, 11.47 +/- 1.96 vs. 12.66 +/- 1.18 yr; p=0.0001) and more advanced puberty at admission (Tanner stage 2, 56.5% vs. 78.1%; p=0.006). Segregation analysis suggested autosomal dominant transmission with incomplete, sex-dependent penetrance (41). Similarly reviewing case histories of familial CPP due to MKRN mutations reveal early and progressive nature of puberty in these girls (42).

SGA-born girls are another special group of children in regard to puberty. Although being born SGA and having catch-up growth is clearly associated with premature pubarche and exagerated premature adrenarche, these children also have accelerated skeletal maturation and tend to have early (not necessarily precocious) but fast puberty resulting in short stature (43).

Finally the risk of developing precocious puberty was significantly increased in adopted girls and in these girls pubertal process usually continue progressively resulting in early menarche, rapid progression of BA and compromised adult height (44,45,46).

### Bone Age-Based Treatment Decision

Some authors suggested predicted height-based decisions regarding GnRHa treatment of girls with CPP. Adan et al (47) used the criteria for treatment as; a PAH <155 cm and/or a LH/FSH peaks ratio of >0.6. Treatment group had greater breast development and BA advances (2.0±0.2 years) and higher plasma estradiol concentrations than the group left untreated. Treated group achieved adult height of 159.5 cm, 3 cm taller than predicted height (156 cm), whereas untreated patients reached an adult height of 162,7 cm, 1.4 cm shorter than predicted height of 164.1 cm. Similarly, Léger et al (22) based treatment decision on BA and LH peak. They did not give treatment in those BA advancement is less than 2 years and peak LH <6 mIU/mL at the initial evaluation. However they decided to begin treatment in girls whose PAH declined during treatment, and were able to achieve a final height better than PAH and surpassing the TH (22).

Thus, BA advancement, and as closely related to it, PAH have major determinants in decision making in regard to GnRHa treatment.

### Handicaps in Bone Age Assessment

BA assessment is one of the key parameters in the management of patient with CPP as it allows the identification of rapidly progressing forms of CPP with compromised PAH, who are thought to benefit most from the treatment in respect to height. Periodical BA evaluation is also a part of monitoring treatment efficacy, as deceleration of BA maturation is expected as a result of treatment. However, BA assessment is affected by a great intra-observer variance, especially around BA of 8-10 years. Nowadays, the majority of girls who are being treated with GnRHa are those between the ages of 6 and 8 years with their BA in the range of 8-10 years.

Although there are several methods for evaluation of BA, the most commonly used method by pediatric endocrinologists is the Greulich-Pyle (GP) method. The GP method is an atlas method in which BA is evaluated by comparing the radiograph of the patient with the nearest standard radiograph in the atlas. Its simplicity, speed and precision made this method the most commonly used standard of reference for skeletal maturation worldwide. However, the GP method was developed using radiographs of upper-middle class Caucasian children in Cleveland, Ohio, United States, and the radiographs were obtained between 1931 and 1942 (48). One has to take the potential insufficiencies of this evaluation into account when evaluating children of today and children from various populations of different ethnic background. Furthermore, these BA methods are based on manual BA determination, the assessment is necessarily subjective and thus, have certain degree of inter-observer and intraobserver difference. In a study, three second year radiology registrars performed both Tanner-Whitehouse 2 (TW2) and GP assessments on each of the BA films. The average spread (the difference between the highest and the lowest of the three results) of BA readings was 0.74 years for TW2 method, and 0.96 years for the GP method (49). Bull et al (50) investigated 362 consecutive “BA” radiographs of the left hand and distal radius performed in a large provincial teaching hospital. Data were analysed using the “method comparison” statistical technique. Ten per cent of the radiographs were re-analysed to assess intra-observer variation. The 95% confidence interval for the difference between the two methods was 2.28 to -1.52 years. Intra-observer variation was greater for the GP method than for the TW2 method (95% confidence limit, -2.46 to 2.18 versus -1.41 to 1.43).

There is now, a recently developed an automated system of BA measurement using computerized image analysis based on both GP and TW2. The use of this automated system was validated in healthy children and in children with various endocrine disorders. It has been shown that automated systems have a better precision and accuracy compared to radiologists’ reading (51). However, still, there are differences in the interpretation of BA, which are big enough to influence clinical decision-making. In a recent study using an automated BA reading the BA difference between the most advanced and most retarded individual bones exceeded 2.0 years. The BA mean differences between the most advanced and most retarded individual bones were 2.58 and 2.25 years for the automated method and GP atlas methods, respectively (52).

### Predicting Height in Girls with ICCP

Height prognosis of the child i.e. “PAH” is of major importance in clinical decision making in girls with CPP. Several alghoritms based on current height and BA to estimate adult height are available but none of them have been fully validated. Predicting adult height with accuracy is hampered by the problems in the accuracy of BA determination as well as problems of methodology in height prediction methods themselves. Bayley-Pinneau method is the most commonly used method for estimating adult height in children have been validated for height prediction in normal children (53). Bayley-Pinneau method estimates adult height as a percentage of current height, based on BA and its relationship to chronological age. It has a wide 95% confidence interval of about 6 cm below to 6 cm above the predicted value, a range which is large enough to mask or blunt small losses or gains in height that occurs due to precocious puberty or its treatment. The prediction equation differs for children whose BA is similar, retarded or advanced in comparison to chronological age (retarded, average and advanced columns in the Bayley-Pinneau height prediction table). Since children with precocious puberty has advanced BA, “advanced column” is used to predict height in girls with CPP. However, it has been shown in several studies that in untreated girls with precocious puberty, Bayley-Pinneau method tend to overrestimate final height by 3.7-5.9 cm in different studies (18,20,23).

To overcome this systematic error it has been proposed that “average column” should be used instead of advanced column (20). This approach might correct the systematic error but is unlikely to increase the precision. Studies reporting PAH in GnRHa treated girls by both advanced and average column demonstrates that final height is closer to PAH calculated with the advanced column than that of the average column (20,26,28,54,55,56). A recent study, when PAH was calculated using the average standards of GP, the median delta final height-PAH was 6.96 and 3.34 cm in GnRHa-treated and nontreated subjects, respectively, whereas when the accelerated standards were used, the differences were less (1.7 and 1.2 cm, p:NS). Final height-PAH-average and final height-PAH-accelerated were comparable among the nontreated subjects but among GnRHa-treated subjects, final height-PAH-average was significantly higher than final height-PAH-accelerated (28). Thus it appears that using advanced column for height prediction gives a better estimation of final height to be reached.

### Height Outcome in Studies with Gonadotropin-Releasing Hormone Analogues Treatment of Progressive Central Precocious Puberty (Table 6)

GnRHa treatment has been a standart of care in girls with progressive CPP for nearly four decades now. GnRHa treatment decreases gonadotrophins, estradiol and the growth velocity and decelerates the skeletal advancement. Linear growth gradually decrease to a rate which is normal for a prepubertal child (~5 cm/year) during the first or second year of treatment, sometimes further deceleration happens in the following years (57,58). Bone maturation also slows down beginning from the 6 months of treatment, averaging 0.5+0.2 BA year/year (59). Similar values have been recorded in other reports (60,61,62). This decrease in bone maturation is progressive and does not occur before six months of treatment (63). As a result of the progressive normalization of BA, and continued linear growth, treatment provides increase in PAH despite the decreased growth velocity, although it is difficult to predict precisely the effect of GnRHa treatment on height gain of these patients, due to handicaps discussed above. Reviewing the available 28 studies on GnRHa treatment of CPP (7,15,16,17,20,22,23,28,47,54,55,56,59,60,61,62,63,64,65,66,67,68,69,70,71,72,73,74) (Table 6) and our own experience (75) demonstrate that the mean age at onset of pubertal development ranged 3-8 years, usually younger and more severe cases in older studies, older and milder cases in recent studies. Nevertheless, in most series, the age of treatment initiation was around 7 years, (5.4-8.7 years) with again recent studies tend to be a year later around 7.5-8 years. Mean BA was around 10 years (8.9-12.5 years) at the beginning of treatment and most series report mean treatment durations around 3.0 years. Mean chronological age at the end of treatment was around 11.1 (9.4-12.7) years of age with a mean BA of 12.4 (11.9-13.6) years. At the achievement of final height all studies except two (69,73) reported final height better than PAH (ranging 2.0-10.5 cm). On average, final height was ~4.0 cm higher than height predicted at the time of initiation of GnRHa treatment.

Comparison of final height of treated patients with their TH eliminates the handicaps of predicting adult heigt thus allows perhaps a better estimation of the effect of GnRHa treatment on height. When compared to TH, in most studies (nineteen studies), final height was 0.4 to 5.2 cm shorter than TH (15,16,20,28,47,54,60,61,62,63,64,65,66,67,68,69,70,71,74) but 0.4-4.2 cm taller in the remaining nine studies (17,22,23,55,56,59,72,73,75). On average, final height was ~1 cm shorter than TH.

However, one should also be aware that, even with comparison with TH is not free of biases. Calculation of midparental TH assumes equal contribution of each parents heights to the offsprings height, thus neglects the effect of dominant genes from one parent. Although TH correlates well with the offsprings height on a population level, it may not correlate well with individual subject. This is especially true for children whose parents are discordant for height.

Finally, in a limited number of studies when adult height of treated patients were compared with untreated study subjects, mean difference ranged from -3.0 to +11 cm) (20,23,28,75). Again, height gain was highly variable among studies depending on sample characteristics including the progression of pubertal development. It should be bear in mind, that the treatment effect also might be overestimated since most of the studies describe observed cases and none of them comprise an intention-to-treat analysis. It is possible that the patients who interrupt the treatment early and are not followed to adult height might have a poorer height outcome than those who continued to the end. Finally, predicted height values obtained during treatment are often overestimated in comparison to the adult height eventually achieved by the patient (7,33).

### Factors Influencing Height Outcome

As mentioned earlier, and seen in the Table 2 data of historical untreated girls with CPP demonstrated that earlier the age of onset of puberty, worse the height prognosis. In line with that, evaluation of treatment series also show that younger age of onset of CPP and hence, younger age of initiation of treatment (which also means longer duration of treatment) is associated with bigger height gain, although a few studies refute that showing no correlation between height gain and age at puberty onset or initiation of treatment (20,59). Greater effectivenesss of GnRHa treatment on younger girls who are destined to poorer height prognosis without treatment, proves further that GnRH treatment is an effective strategy to preserve diminished height potential in these children.

BA advance at start of treatment and at the end, is negatively associated with height outcome (7,47,54,65,73). BA/statural age ratio at the onset of treatment and adult height is negatively associated with outcome suggesting that treatment is not capable of restoring a full adult height potential if started after a certain critical advancement of BA. Kauli et al (20) demonstrated that therapy is more beneficial if started before BA exceeds 12 years.

Height SDS at the onset (7,17,33,54,56,62,67) and at termination of treatment (7,17,54,56,59,67,73), as well as higher TH (7,17,65,67,72) have also been positively associated with adult height, supporting that influence of genetic factors on height is dominant among other factors.

Naturally, BA at the end of treatment, is associated with final height, as it determines posttreatment residual growth potential (7,59,71). Although data are scarce in this respect, stopping treatment at a BA of 12-12.5 years (7) or even <11.5 years (26) seemed to be associated with best height outcome, while continuing treatment after a BA ≥13 years negatively impacted on statural growth (7). Three factors explained 66% of adult height variance: BA advance before treatment, height at the end of treatment and height gain after interruption of treatment (33).

In summary, among the factors associated with the height outcome, height SDS and TH reflecting genetic potential, are always associated with positive outcome, BA advance and delay in treatment are negative factors. This highlights the importance of rapid recognition, evaluation and treatment of patients with true precocious puberty. However, one has to balance this with careful follow-up in some girls to not treat those with slow progression unnecessarily.

In terms of effcicacy of treatment, various GnRHa appeared similar as regards to height outcome (26,62,71,74), except for a study (69) demonstrating slightly better adult height SDS in patients treated with leuprolide depot compared to triptorelin depot.

### Optimal Age of Discontinuation of Gonadotropin-Releasing Hormone Agonist Treatment in Girls with Central Precocious Puberty

Data is also missing on this respect. In the literature, (Table 6) the mean age at interruption of treatment ranges 9.4 to 12.7 averaging around age 11 year and BA ranging from 11.9 to 13.6 averaging 12.5 years. BA at the end of treatment correlates negatively with height gain after treatment. Carel et al (33) using multivariate analysis estimated that an 11 year old girl, growing 4 cm and gaining 0.5 BA year per year, could loose 2.6 cm of adult height if treatment was discontinued 1 year later. Opposite results were found by Klein et al (62) who found a positive correlation between age at discontinuation of treatment and adult height (r=0.25, p=0.03), suggesting that prolonging the treatment could increase height. Obviously, this discrepancy only can be solved with a formal controlled trial (i.e. randomizing girls between “early” and “late” age at discontinuation of treatment). However, such a trial would be difficult to perform since patients and the parents prefer to stop treatment when the girl has reached an age that peers of the patients have already started puberty which is usually around age 11 year.

Could the BA be a useful parameter to decide when to stop treatment? Although the optimal age for treatment interruption is not clearly defined by international guidelines, it has been proposed that the best heights are achieved when treatment is discontinued at around 12-12.5 years in girls (7,76) However, in girls around the age of 11 years with previous advance in BA and a long-standing treatment with GnRH agonists, BA often is approximately 12 years with little variation and is therefore of little help to orient decisions. Furthermore, reduction of growth velocity, commonly observed around this age, due to the increasing dependence of growth on sex steroids (77) with time, necessitates stopping treatment.

### Treatment of Gonadotropin-Releasing Hormone Analogues Combined with Growth Hormone Treatment (Table 7)

The growth velocity in some CPP patients decreases below the normal for prepubertal children during GnRHa therapy. Subnormal growth velocity during GnRHa therapy may be associated with a decrease in GH and insulin-like growth factor 1 secretion due to suppression of gonadal steroids (77). Therefore, some studies investigated in girls with precocious puberty and poor predicted height, whether adding GH to GnRHa treatment is associated with a better height outcome (78,79,80,81,82,83,84). Data is even more limited and biased about this type of approach. In short, it can be stated that at present, studies are insufficient to make definite conclusions about the height outcomes of GnRHa plus GH treatment. Lanes and Gunczler (78) treated 15 short children (boys and girls) entering into normally timed puberty with both GnRHa and GH and compared them with an identical number of untreated children. In their study, no relevant height gain was observed after 2.5 years of treatment.

Pasquino et al (79) and Pucarelli et al (80) on the other hand, showed differences of about 6-8 cm of height gain on girls with CPP treated with GnRHa plus GH, compared to GnRHa alone. In their first report, the gain in centimeters, (calculated between pretreatment PAH (152.7±1.7 cm) and final height (160.6±1.3 cm), was 7.9±1.1 in patients treated with GH plus GnRHa, whereas in patients treated with GnRHa alone, the gain between pretreatment PAH (155.5±1.7) and final height (157.1±2.5 cm) was just 1.6 cm ± 1.2. The difference between the gain obtained in the groups is significant, in favor of combination group (p<0.001) (79). However, the same group reported four years later a larger number of patients with a longer follow-up period that, adult height versus pre-treatment PAH was 6 cm greater in combination treatment than that of GnRHa alone but concluded that true efficacy of the addition of GH to GnRHa therapy is still questionable (80). They recommended caution regarding such an invasive and expensive treatment, outside a research setting.

It should also be taken into account that, in the above studies, the treatment period was not standardized, and the authors treated a selected group of patients, i.e. those whose height velocity decreased to value <p25 for chronological age under GnRHa treatment. Besides, the duration of treatment in these studies was remarkably longer than in other studies with combined treatment and GH dosage was higher.

A randomized controlled study, in short adopted girls with early puberty, Mul et al (81) treated girls with onset of puberty before 10 years of age for 3 years with either GnRHa alone (group A, n=12) or with GnRHa and GH (group B, n=14). Height gain defined as the difference between initial height prediction and attained final height, was significantly different between group A and B (5.2±3.7 cm and 8.2±3.4 cm, p<0.05) using average tables for height prediction. However, with advanced tables for height prediction, the numbers were much less (-1.0±3.6 and 3.3±3.5 cm, respectively).

A recent Korean study in 82 girls with idiopathic CPP showed a height gain of approximately 3.8 cm in the GnRHa alone group, while 4.7 cm in the combination group compared to PAH before treatment with no statistically significant difference between two groups. (83). Finally a recent meta-analyses, evaluating a total of six randomized controlled trials (RCTs) (162 patients) and six clinical controlled trials (CCTs) (247 patients) reported that compared to the GnRHa therapy group, the combination therapy group achieved taller final height (mean difference=2.81 cm, four CCTs and 4.30 cm, in one RCT); and 3.9 cm better final height compared with THs (84).

The results of these studies (comparing adult height vs. predicted height) should again be interpreted in the context of the before mentioned methodological handicaps of accurately predicting adult height. Furthermore, the number of treated patients are much less, and most likely involves selection biase as those who have poor height potential or attenuated growth velocity might tend to choose or given the combined GnRHa GH treatment. Finally, since GH treatment requires the consideration of cost, economic status may be another affecting factor to select the patients treated with GnRHa plus GH. Cost-effectiveness of combined GH treatment in patients with CPP has also to be elucidated.

## Figures and Tables

**Table 1:**
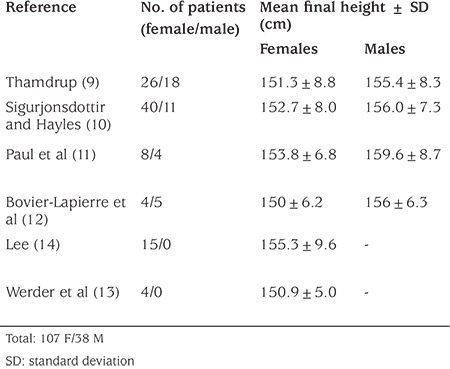
Historical data of untreated children with precocious puberty

**Table 2:**
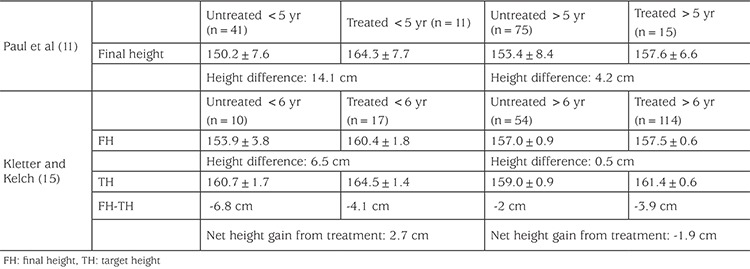
Adult heights (cm) of treated and untreated (historical) girls with central precocious puberty according to the age of onset

**Table 3:**
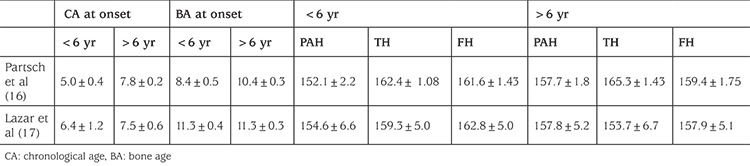
Effect of age of onset of treatment on height (studies with no control group)

**Table 4:**
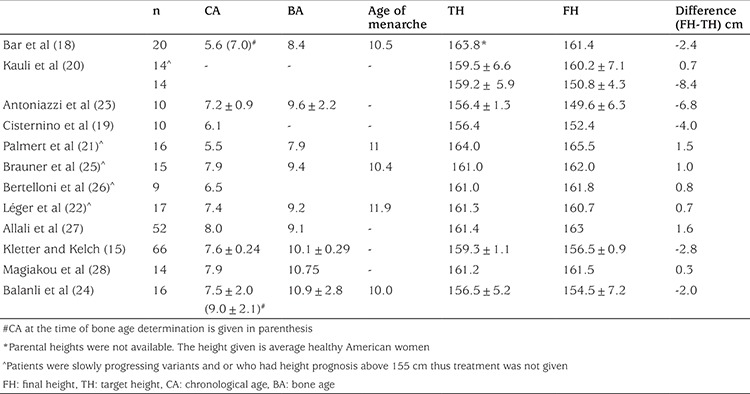
Final height of girls with untreated central precocious puberty (slowly progressive, milder, or older onset) patients in different series

**Table 5:**
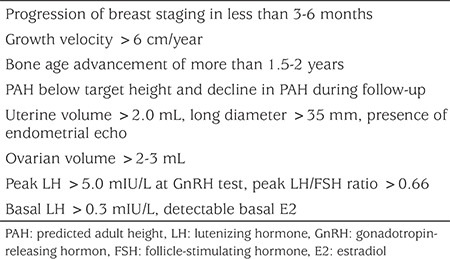
Criteria for identifying girls who are likely to have progressive precocious puberty

**Table 6:**
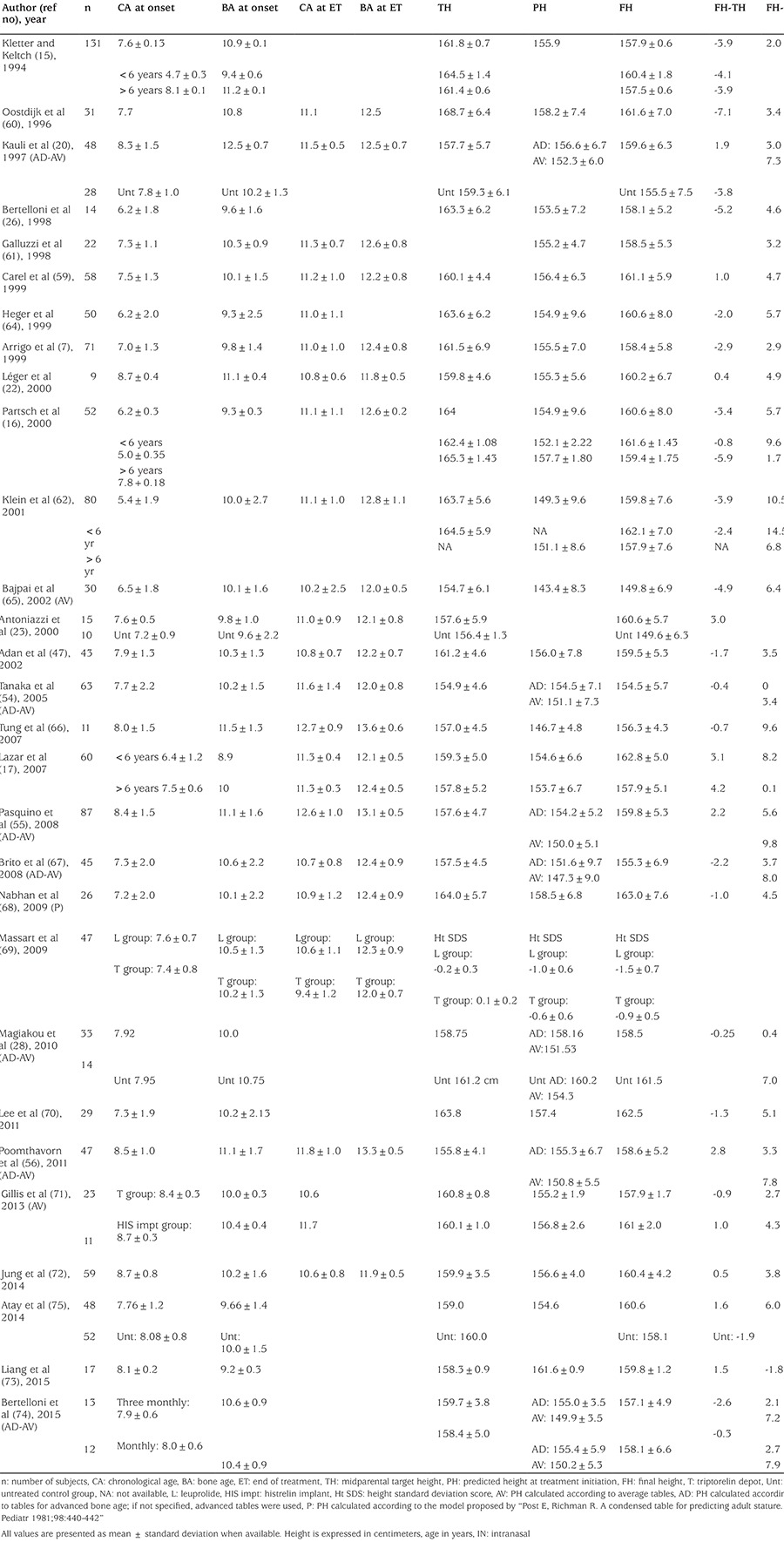
Height outcome in studies with gonadotrophin‐releasing hormone agonists treatment of progressive idiopathic central precocious puberty

**Table 7:**
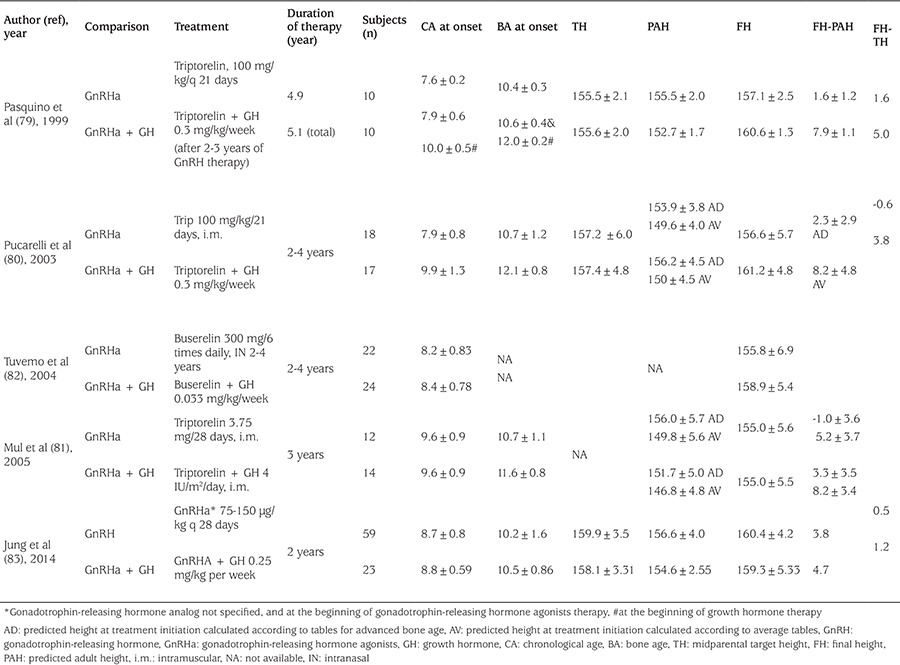
Studies investigating gonadotrophin‐releasing hormone analog plus growth hormone treatment on final height of girls with central precocious puberty
